# Pilot Study to Compare the Use of End‐Tidal Carbon Dioxide–Guided and Diastolic Blood Pressure–Guided Chest Compression Delivery in a Swine Model of Neonatal Asphyxial Cardiac Arrest

**DOI:** 10.1161/JAHA.118.009728

**Published:** 2018-09-27

**Authors:** Caitlin E. O'Brien, Michael Reyes, Polan T. Santos, Sophia E. Heitmiller, Ewa Kulikowicz, Sapna R. Kudchadkar, Jennifer K. Lee, Elizabeth A. Hunt, Raymond C. Koehler, Donald H. Shaffner

**Affiliations:** ^1^ Department of Anesthesiology/Critical Care Medicine Johns Hopkins University School of Medicine Baltimore MD; ^2^ Department of Pediatrics Johns Hopkins University School of Medicine Baltimore MD; ^3^ Department of Physical Medicine & Rehabilitation Johns Hopkins University School of Medicine Baltimore MD; ^4^ Division of Health Sciences Informatics Johns Hopkins University School of Medicine Baltimore MD

**Keywords:** capnography, cardiopulmonary resuscitation, diastolic blood pressure, pediatrics, physiologic feedback, Animal Models of Human Disease, Cardiopulmonary Arrest, Cardiopulmonary Resuscitation and Emergency Cardiac Care, Hemodynamics

## Abstract

**Background:**

The American Heart Association recommends use of physiologic feedback when available to optimize chest compression delivery. We compared hemodynamic parameters during cardiopulmonary resuscitation in which either end‐tidal carbon dioxide (ETCO_2_) or diastolic blood pressure (DBP) levels were used to guide chest compression delivery after asphyxial cardiac arrest.

**Methods and Results:**

One‐ to 2‐week‐old swine underwent a 17‐minute asphyxial‐fibrillatory cardiac arrest followed by alternating 2‐minute periods of ETCO_2_‐guided and DBP‐guided chest compressions during 10 minutes of basic life support and 10 minutes of advanced life support. Ten animals underwent resuscitation. We found significant changes to ETCO_2_ and DBP levels within 30 s of switching chest compression delivery methods. The overall mean ETCO_2_ level was greater during ETCO_2_‐guided cardiopulmonary resuscitation (26.4±5.6 versus 22.5±5.2 mm Hg; *P*=0.003), whereas the overall mean DBP was greater during DBP‐guided cardiopulmonary resuscitation (13.9±2.3 versus 9.4±2.6 mm Hg; *P*=0.003). ETCO_2_‐guided chest compressions resulted in a faster compression rate (149±3 versus 120±5 compressions/min; *P*=0.0001) and a higher intracranial pressure (21.7±2.3 versus 16.0±1.1 mm Hg; *P*=0.002). DBP‐guided chest compressions were associated with a higher myocardial perfusion pressure (6.0±2.8 versus 2.4±3.2; *P*=0.02) and cerebral perfusion pressure (9.0±3.0 versus 5.5±4.3; *P*=0.047).

**Conclusions:**

Using the ETCO_2_ or DBP level to optimize chest compression delivery results in physiologic changes that are method‐specific and occur within 30 s. Additional studies are needed to develop protocols for the use of these potentially conflicting physiologic targets to improve outcomes of prolonged cardiopulmonary resuscitation.

**Clinical Perspective**

**What Is New?**

- During cardiopulmonary resuscitation after asphyxial cardiac arrest, chest compression delivery can be quickly altered to optimize end‐tidal carbon dioxide or diastolic blood pressure.
- These 2 methods of real‐time physiologic feedback produce unique changes in clinically important resuscitation variables.
- End‐tidal carbon dioxide–guided chest compression delivery results in a faster compression rate and higher intracranial pressure, whereas diastolic blood pressure–guided chest compressions are associated with higher myocardial and cerebral perfusion pressures.

**What Are the Clinical Implications?**

- The use of end‐tidal carbon dioxide or diastolic blood pressure level to guide chest compression delivery results in hemodynamic changes that are method‐specific and warrants further study before the development of personalized resuscitation protocols.

## Introduction

Cardiac arrest occurs in 2% of patients admitted to the pediatric intensive care unit.^1^, ^2^ Neurologically intact survival after in‐hospital pediatric cardiac arrest has improved since 2000,^3^ but mortality (35%–67%) and unfavorable neurologic outcome (17%–53% of survivors) remain high.^2^, ^4^, ^5^, ^6^, ^7^, ^8^ Use of physiologic feedback to guide and personalize resuscitative efforts may be a strategy to improve outcome.

The American Heart Association (AHA) recommends the use of physiologic feedback, including end‐tidal CO_2_ (ETCO_2_) and invasive hemodynamic monitoring, when available, to optimize resuscitation.^9^ Studies are still needed to determine which physiologic feedback techniques are most beneficial. Because of its continuous real‐time availability, ETCO_2_ has great potential for use as physiologic feedback during cardiopulmonary resuscitation (CPR). ETCO_2_ is a noninvasive marker of pulmonary blood flow and linearly correlates with cardiac output during low‐flow states, including during CPR.^10^, ^11^, ^12^, ^13^, ^14^, ^15^ Higher initial and mean ETCO_2_ levels during CPR are associated with return of spontaneous circulation (ROSC) and survival.^16^, ^17^, ^18^, ^19^ Use of ETCO_2_ to monitor quality of CPR in adults is associated with improved rates of ROSC.^20^ We have developed an ETCO_2_‐guided chest compression delivery model that was shown to increase the rate of ROSC after prolonged asphyxial cardiac arrest in neonatal swine.^21^ This model can be used as a comparator for other methods of physiologic feedback to gain further understanding of their applications.

Myocardial perfusion during CPR occurs during the relaxation (diastolic) phase of chest compression delivery and must be adequate to achieve ROSC. Survival in both preclinical and clinical studies of cardiac arrest is associated with increased relaxation/diastolic blood pressure (DBP) and/or myocardial perfusion pressure (MPP) during CPR.^22^, ^23^, ^24^, ^25^, ^26^, ^27^ A method of hemodynamic‐directed CPR that used systolic pressure to guide chest compression delivery and diastolic pressure to guide epinephrine delivery improved survival in preclinical models.^28^, ^29^, ^30^, ^31^, ^32^

To our knowledge, the use of DBP‐guided chest compression delivery has not been described. In this study, we compared our previous method of ETCO_2_‐guided chest compression delivery with DBP‐guided chest compression delivery. Our primary objective was to characterize and compare chest compression delivery and physiologic parameters using discrete epochs of ETCO_2_‐guided resuscitation alternated with DBP‐guided resuscitation in a neonatal swine model of prolonged asphyxial‐fibrillatory cardiac arrest.

## Methods

All data and materials have been made publicly available at the Open Science Framework and can be accessed at DOI 10.17605/osf.io/uf984.

### Model Justification

We chose swine because the size and shape of the thorax is comparable to that of a neonate and allows CPR delivery using the 2‐thumb‐encircling hands technique. Moreover, the 2‐week‐old piglet approximates a neonate's size and weight of 3 to 4 kg. In adult swine, the chest wall mechanics during CPR compare favorably with those of humans.^33^ We chose an asphyxial model because the leading cause of pediatric cardiac arrest is respiratory compromise.^5^, ^6^

### Animal Preparation

All studies were carried out with approval by the Animal Care and Use Committee at the Johns Hopkins University in accordance with institutional guidelines. The protocol used was similar to those we have described previously.^21^, ^34^ Ten male Yorkshire swine (7–14 days old, 3–4 kg) received anesthesia with 2% isoflurane, 70% nitrous oxide, and 30% oxygen. A 4.0 cuffed tracheal tube was secured via tracheostomy. A femoral venous catheter was advanced to the midthoracic vena cava for administration of sedation and epinephrine, and for measurement of central venous pressure (CVP). A femoral artery catheter was advanced to the midthoracic aorta for continuous hemodynamic monitoring and blood gas determinations. A pacing wire was placed through the other femoral vein and advanced until ventricular irritation confirmed right ventricular placement. A sagittal sinus catheter was placed through a burr hole at the bregma to measure intracranial pressure (ICP) and to obtain venous blood gases. The anteroposterior chest diameter was measured before cardiac arrest and at the end of the experiment to determine compression‐induced chest wall deformity. After the surgery was complete, fentanyl (10 μg/kg) and vecuronium (0.3 mg/kg) were administered and isoflurane was discontinued. Additional doses of fentanyl were administered as needed during postsurgical stabilization. Rectal temperature was maintained at the normal swine core temperature of 38°C to 39°C with a heating pad. Pressure‐controlled ventilation was adjusted to maintain a Paco_2_ of 35 to 45 mm Hg at a rate of 20 breaths per minute during surgery and stabilization. We replaced the nitrous oxide with room air and decreased the FiO_2_ from 0.30 to 0.21 to ensure normoxia before initiating cardiac arrest. Once the asphyxia began, no further anesthetics were administered. Vital signs, including ETCO_2_, DBP, mean arterial blood pressure (MAP), CVP, ICP, heart rate or compression rate, and rectal temperature were recorded before protocol initiation (baseline) and at 30‐s intervals during asphyxia and resuscitation.

### Experimental Protocol

**Figure 1:**
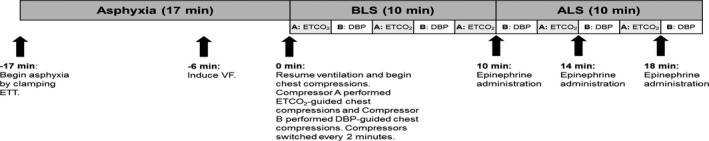
Experimental timeline in minutes. ALS indicates advanced life support; BLS, basic life support; ETCO_2_, end‐tidal carbon dioxide; ETT, endotracheal tube; DBP, diastolic blood pressure; VF, ventricular fibrillation.

A timeline of the experimental protocol is provided in Figure 1. We produced a 17‐minute asphyxial‐fibrillatory arrest by clamping the endotracheal tube for 11 minutes and then inducing 6 minutes of ventricular fibrillation by applying 50 mA of alternating current via the femoral pacing wire. We added fibrillation to prevent ROSC from interfering with the planned 20 minutes of physiologic data collection during CPR. We chose to fibrillate at 11 minutes because most piglets had become pulseless and the interval is sufficiently brief that the ventricular fibrillation produced is coarse and easy to confirm on an ECG. After 17 minutes of asphyxia, pediatric basic life support (BLS) with chest compression and mechanical ventilation was initiated. Mechanical ventilation was provided with an FiO_2_ of 1.0 at the pre‐arrest rate of 20 beats per minute and the pre‐arrest inspiratory pressure with pressure‐controlled ventilation to maintain normoxia and normocapnia as documented on baseline laboratory evaluation. The ventilation rate of 20 breaths per minute is higher than that recommended by the AHA, but in this model, we have found that the higher rate offsets the reduced tidal volumes produced when using pressure‐limited ventilation during resuscitation. Chest compressions were performed with a 2‐thumb‐encircling hands technique, and compressors switched every 2 minutes as per AHA guidelines.^9^ A coach critiqued the compressors in real time to limit leaning and incomplete chest recoil during resuscitation.

During the 2‐minute intervals of ETCO_2_‐guided chest compression delivery, Compressor A maximized ETCO_2_ to the highest obtainable value (no specific numeric goal) by changing the chest compression rate, force, depth, and thumb positioning. Compressor A was blinded to DBP, MAP, CVP, and ICP. During the 2‐minute intervals of DBP‐guided chest compression delivery, Compressor B maximized DBP to the highest obtainable value (no specific numeric goal) by changing the chest compression rate, force, depth, and thumb positioning. Compressor B was blinded to ETCO_2_, MAP, CVP, and ICP. The sequence of Compressor A delivering 2 minutes of ETCO_2_‐guided CPR followed by Compressor B delivering 2 minutes of DBP‐guided CPR was repeated for 5 periods to total 20 minutes of CPR (Figure 1).

To compare the effects of BLS and advanced life support (ALS) on the 2 chest compression methods, we included 10 minutes of BLS (no epinephrine administration) followed by 10 minutes of ALS (with epinephrine administration) in the 20 minutes of CPR. During ALS, epinephrine (300 μg) was administered via the midthoracic venous catheter at 10, 14, and 18 minutes of CPR (Figure 1). Each dose was followed by a 10‐mL normal saline bolus to ensure delivery and circulation. Because our goal was to obtain physiologic data throughout the entire resuscitation, no attempts at defibrillation were made. Physiologic data were collected at baseline and at 30‐s intervals during asphyxia and resuscitation. Arterial and venous blood gases were examined at baseline, 10 minutes of asphyxia, and 8 and 20 minutes of CPR. We performed autopsies on all animals to determine resuscitation‐related injuries, including superficial liver injury, superficial cardiac injury, atelectasis, and hemothorax.

### Statistical Analysis

Physiologic data are presented as mean±SEM. The MPP was calculated as DBP minus diastolic CVP. Systemic perfusion pressure was calculated as MAP minus mean CVP. Cerebral perfusion pressure (CPP) was calculated as MAP minus the greater of ICP or mean CVP. Because the ICP was almost universally higher, it was used for standardization of CPP. Differences in physiologic data during ETCO_2_‐guided and DBP‐guided chest compression delivery were evaluated by paired *t* test of averages during BLS, ALS, and the entire resuscitation. Two‐tailed *P*≤0.05 were considered significant. All data were analyzed in Stata (Version 14; StataCorp LLC, College Station, TX).

Data were analyzed during 3 distinct timeframes, including BLS, ALS, and the entire 20‐minute resuscitation. Data analysis included all 4 data points during each 2‐minute epoch. We excluded data from the first minute of CPR (which represented recovery from hypercarbia and falsely elevated ETCO_2_) and from the first minute of ALS (which represented initial epinephrine administration and not hemodynamics produced by the CPR method).

## Results

Ten animals underwent cardiac arrest and resuscitation. Data collection was complete with the following exceptions. The sagittal venous catheter malfunctioned midexperiment in 1 animal, preventing collection of venous blood gas data after the baseline. In another animal, the arterial line malfunctioned and the experiment was terminated early after 14 minutes of resuscitation. Data for the first 14 minutes of resuscitation were included in the analyses. Finally, diastolic CVP was not recorded for 2 animals, preventing calculation of MPP. Therefore, diastolic CVP and MPP data were available for only 8 animals, whereas all other data were available for 10 animals.

Physiologic data at baseline (pre‐arrest) are shown in Table 1. Baseline data were within normal limits for swine. Clamping the endotracheal tube for asphyxia produced a mean arterial pH of 6.84±0.04, a Paco_2_ of 126±5 mm Hg, and a Pao_2_ of 10±2 mm Hg after 10 minutes. Eight minutes of resuscitation with chest compressions and mechanical ventilation improved arterial pH to 7.11±0.04, Paco_2_ to 36±6 mm Hg, and Pao_2_ to 147±28 mm Hg. At 20 minutes of CPR, the arterial pH was 6.95±0.03, Paco_2_ was 48±7 mm Hg, and Pao_2_ was 155±28 mm Hg.

**Table 1:** Baseline Characteristics

Parameter	All (n=10)
Weight, kg	3.74±0.05
HR, bpm	218±15
ETCO_2_, mm Hg	47±2
DBP, mm Hg	72±5
MAP, mm Hg	87±5
dCVP^a^, mm Hg	6.6±0.6
mCVP, mm Hg	7.4±0.5
MPP^a^, mm Hg	66±6
SPP, mm Hg	80±5
ICP, mm Hg	11±1
CPP, mm Hg	76±6
Temperature, °C	38.7±0.2
pHa	7.37±0.01
pHv	7.31±0.02
Paco_2_, mm Hg	43±1
Pvco_2_, mm Hg	50±2
Pao_2_, mm Hg	89±6
Pvo_2_, mm Hg	34±4
BEa	−0.5±0.8
BEv	−0.7±1.2
Sao_2_, %	94±2
Svo_2_, %	50±6
Hb, g/dL	10.7±0.4

Data were collected before asphyxia and are shown as mean± SEM. BEa indicates base excess arterial; BEv, base excess venous; bpm, beats per minute; CPP, cerebral perfusion pressure; DBP, diastolic blood pressure; dCVP, diastolic central venous pressure; ETCO_2_, end‐tidal carbon dioxide; Hb, hemoglobin; HR, heart rate; ICP, intracranial pressure; MAP, mean arterial blood pressure; mCVP, mean central venous pressure; MPP, myocardial perfusion pressure; Paco_2_, arterial carbon dioxide partial pressure; Pao_2_, arterial oxygen partial pressure; pHa, arterial pH; pHv, venous pH; Pvco_2_, venous carbon dioxide partial pressure; Pvo_2_, venous oxygen partial pressure; Sao_2_, arterial oxygen saturation; SPP, systemic perfusion pressure; Svo_2_, venous oxygen saturation.

a Data were available for only 8 animals.

**Figure 2:**
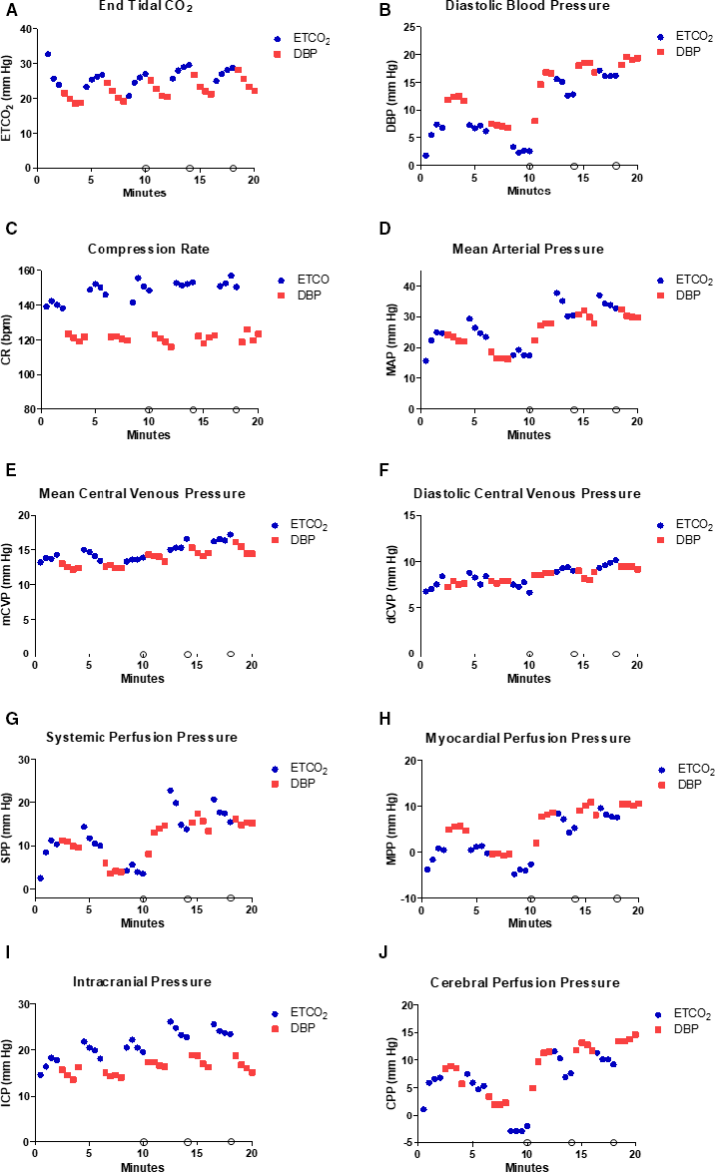
Hemodynamic variables during cardiopulmonary resuscitation. Hemodynamic variables were measured during basic life support (min 0–10) and advanced life support (min 10.5–20) as CPR was delivered with ETCO_2_‐guided chest compression (blue circles) and DBP‐guided chest compression (red squares). Each data point represents the mean value at 30‐s intervals. Open circles along the *x*‐axis represent epinephrine administration at 10, 14, and 18 min of CPR. A, End‐tidal CO_2_ (ETCO_2_). B, Diastolic blood pressure (DBP). C, Compression rate (CR) in beats per minute (bpm). D, Mean arterial pressure (MAP). E, Mean central venous pressure (mCVP). F, Diastolic central venous pressure (dCVP). G, Systemic perfusion pressure (SPP). H, Myocardial perfusion pressure (MPP). I, Mean intracranial pressure (ICP). J, Cerebral perfusion pressure (CPP).

At the initiation of CPR, ETCO_2_ exceeded the upper limit of detection by capnography (99 mm Hg, not shown). By 30 s of CPR, the mean ETCO_2_ had declined to 57±4 mm Hg (not shown), and at 1 minute it was 33±4 mm Hg (Figure 2A and Figure S1A). The ETCO_2_ level was significantly higher during ETCO_2_‐guided chest compressions than during DBP‐guided chest compressions over the entire resuscitation (26.4±5.6 versus 22.5±5.2 mm Hg; *P*=0.003; Table 2, excluding the first minute of CPR). Mean ETCO_2_ was also significantly higher with ETCO_2_‐guided than with DBP‐guided chest compressions during both BLS (*P*<0.0001) and ALS (*P*=0.0008; Table 2). The changes in ETCO_2_ level were consistent during chest compression delivery optimizing ETCO_2_ (Figure 2A and Figure S1A). The improvement in mean ETCO_2_ level produced by ETCO_2_‐guided chest compressions during the 4 to 6‐, 8 to 10‐, 12 to 14‐, and 16 to 18‐minute periods was 8.2±1.7 mm Hg (8.0±1.6, 7.9±1.9, 9.1±1.5, and 7.5±1.9 mm Hg, respectively). Approximately half of the improvement in mean ETCO_2_ occurred during the first 30 s of ETCO_2_‐guided CPR in each of the 4 periods analyzed (3.8±1.1 mm Hg). These improvements in the ETCO_2_ level during ETCO_2_‐guided chest compression were unchanged by the switch from BLS to ALS. The compression rate was significantly greater during ETCO_2_ guidance than during DBP‐guided CPR (149±3/min versus 120±5/min; *P*=0.0001; Table 2). The average increase in compression rate to produce the increase in ETCO_2_ was 29±5 compressions/min (Table 2). Intracranial pressure was greater during ETCO_2_ guidance than during DBP guidance (*P*=0.002; Table 2). The increases in compression rate and mean ICP were apparent during both BLS and ALS. MAP was significantly greater with ETCO_2_ guidance during ALS (*P*=0.03; Table 2). Systemic perfusion pressure, MAP, and mean CVP did not differ significantly between ETCO_2_ and DBP guidance (Table 2).

**Table 2:** Hemodynamic Results for the Entire Resuscitation, Basic Life Support and Advanced Life Support

Parameter	ETCO_2_‐Guided (n=10)	DBP‐Guided (n=10)	*P* Value
Entire resuscitation			
ETCO_2_, mm Hg	26.4±5.6	22.5±5.2	0.003
DBP, mm Hg	9.4±2.6	13.9±2.3	0.003
Comp/min	149±3	120±5	0.0001
MAP, mm Hg	27.3±3.6	24.9±3.0	0.08
dCVP^a^, mm Hg	8.4±0.6	8.3±0.4	0.63
mCVP, mm Hg	14.8±1.3	13.5±0.9	0.06
MPP^a^, mm Hg	2.4±3.2	6.0±2.8	0.02
SPP, mm Hg	12.5±3.8	11.4±2.9	0.41
ICP, mm Hg	21.7±2.3	16.0±1.1	0.002
CPP, mm Hg	5.5±4.3	9.0±3.0	0.047
Basic life support (0–10 min)			
ETCO_2_, mm Hg	25.0±5.4	20.6±5.2	<0.0001
DBP, mm Hg	5.3±2.4	9.6±2.0	0.004
Comp/min	147±3	121±5	0.002
MAP, mm Hg	22.5±3.1	19.9±2.6	0.11
dCVP^a^, mm Hg	7.8±0.8	7.7±0.4	0.85
mCVP, mm Hg	14.0±1.2	12.5±0.7	0.045
MPP^a^, mm Hg	−1.1±3.1	2.4±2.5	0.04
SPP, mm Hg	8.5±3.2	7.4±2.6	0.39
ICP, mm Hg	19.9±2.3	14.8±1.2	0.004
CPP, mm Hg	2.6±3.8	5.2±2.8	0.11
Advanced life support (10.5–20 min)			
ETCO_2_, mm Hg	28.6±6.1	24.5±5.5	0.0008
DBP, mm Hg	15.3±3.0	18.4±2.6	0.02
Comp/min	152±4	121±4	<0.0001
MAP, mm Hg	34.3±4.6	30.5±3.5	0.03
dCVP^a^, mm Hg	9.3±0.5	8.8±0.4	0.054
mCVP, mm Hg	15.8±1.4	14.3±1.0	0.04
MPP^a^, mm Hg	7.3±3.7	10.0±3.2	0.02
SPP, mm Hg	18.5±4.9	16.2±3.6	0.19
ICP, mm Hg	24.4±2.3	17.2±1.1	0.0007
CPP, mm Hg	9.9±5.0	13.4±3.4	0.11

Data represent the cumulative 30‐s values during the entire 20 min of cardiopulmonary resuscitation, the 10 min of basic life support, or the 10 min of advanced life support and are shown as mean±SEM. Comp/min indicates compressions per minute; CPP, cerebral perfusion pressure; DBP, diastolic blood pressure; dCVP, diastolic central venous pressure; ETCO_2_, end‐tidal carbon dioxide; ICP, intracranial pressure; MAP, mean arterial blood pressure; mCVP, mean central venous pressure; MPP, myocardial perfusion pressure; SPP, systemic perfusion pressure.

a Data were available for only 8 animals.

DBP increased during DBP‐guided chest compression delivery and was greater than that achieved with ETCO_2_ guidance during both BLS and ALS (*P*=0.004 and *P*=0.02, respectively). Mean DBP was 13.9±2.3 mm Hg during DBP‐guided CPR and 9.4±2.6 mm Hg during ETCO_2_‐guided CPR (*P*=0.003; Table 2) over the entire 20 minutes of resuscitation (excluding minute 10 to 11 of CPR after the initial epinephrine dose was administered). The improvement in mean DBP produced by DBP‐guided chest compressions during the 2 to 4‐, 6 to 8‐, 14 to 16‐, and 18 to 20‐minute epochs was 2.8±2.1 mm Hg (4.9±1.5, 0.6±2.4, 2.3±2.8, and 3.1±1.2 mm Hg, respectively). We excluded the period from 10 to 12 minutes because of the dramatic effect of the initial epinephrine dose. The improvement in DBP with DBP‐guided chest compression was similar during BLS and ALS, but DBP was improved with both methods during ALS after the initiation of epinephrine (Figure 2B and Figure S1B). All the increase in DBP occurred in the first 30 s after the change to DBP‐guided chest compression delivery (3.5±1.6 mm Hg). During DBP‐guided chest compressions, MPP increased significantly (6.0±2.8 versus 2.4±3.2 mm Hg; *P*=0.02; Table 2). This elevation in MPP was present during both BLS and ALS (*P*=0.04 and *P*=0.02, respectively). DBP‐guided chest compression delivery also produced an increase in CPP (9.0±3.0 versus 5.5±4.3 mm Hg; *P*=0.047; Table 2).

Autopsy findings were not different from those of our previous studies with experimental or standard CPR.^21^, ^34^ The average chest deformity (change in pre‐arrest to post‐resuscitation anteroposterior chest diameter) was 1.6±0.2 cm (14% of baseline diameter). Nine animals (90%) developed atelectasis, 6 (60%) developed epicardial hemorrhages along the anterior surface parallel to the coronary artery, 1 (10%) developed a hemothorax, and no animal developed superficial liver laceration.

## Discussion

This study provides an initial description of the use of DBP to optimize chest compression delivery and is the first, to our knowledge, to compare the use of ETCO_2_ and DBP as real‐time physiologic feedback for chest compression guidance during CPR in a neonatal animal model. By alternating between these 2 methods of guided chest compression delivery, we showed (1) that each produces effects on clinically important resuscitation variables, (2) that chest compression delivery can be optimized to quickly improve the targeted physiologic variable, and (3) that changes to important hemodynamic variables during CPR vary by the guidance method used. We used the ETCO_2_ level during CPR as 1 physiologic target for chest compression delivery because it represents the blood flow through the lungs and is a surrogate for the systemic perfusion produced by resuscitative efforts.^11^ We compared it with DBP as a physiologic target for chest compression delivery because DBP is a surrogate for the myocardial perfusion produced by resuscitative efforts and is critical to achieving ROSC.^22^, ^26^ The finding that these 2 chest compression methods can produce rapid, clinically relevant effects on important resuscitation variables indicates their potential to individualize and optimize chest compression delivery during prolonged resuscitation.

As previously shown, ETCO_2_ is not an accurate guide for chest compression delivery immediately after asphyxia, but by 1 minute of resuscitation it decreases to a reliable level that can be used for feedback.^35^, ^36^ Figure 2A illustrates that large and consistent changes in the ETCO_2_ level were possible during the last 4 periods of ETCO_2_‐guided chest compression delivery. The ETCO_2_ level during ETCO_2_‐guided chest compression delivery increased by ≈8 mm Hg (range, 7.5–9.1 mm Hg) without influence by the transition from BLS to ALS. These increases in ETCO_2_ were likely because of an increase in compression rate (Table 2 and Figure 2C) and force (inferred by increases in MAP and ICP; Table 2 and Figure 2D and 2I). This finding is consistent with our previous work in which ETCO_2_‐guided chest compression delivery resulted in a chest compression rate of 143±10/min.^21^ In that study, ETCO_2_‐guided CPR resulted in an ETCO_2_ level >30 mm Hg over 20 minutes of CPR despite a more significant injury (20 minutes of asphyxia).^21^ In the current study, ETCO_2_ levels did not reach >30 mm Hg, probably because we limited each compression method to 2‐minute epochs. In the previous study, we found that ETCO_2_‐guided chest compression delivery improved DBP, CPP, systemic perfusion pressure, and achievement of ROSC compared with 20 minutes of standard CPR (no physiologic feedback). Our experience is that ETCO_2_‐guided chest compression delivery is an easily applied physiologic feedback method when invasive monitoring is not available. Because ETCO_2_‐guided chest compression delivery appears to enhance systemic perfusion more than myocardial perfusion,^21^ it is important to understand how this method compares with techniques intended to improve myocardial perfusion, such as DBP‐guided CPR.

Several swine studies have shown that the use of physiologic feedback to provide hemodynamic‐directed CPR improves myocardial perfusion and outcome compared with standard CPR.^28^, ^29^, ^30^, ^31^, ^32^ The hemodynamic‐directed CPR described in those studies used systolic blood pressure as the target for chest compression depth and coronary perfusion pressure as the target for epinephrine administration. A technique that alters chest compression delivery to maximize DBP may not be as effective as one that alters the epinephrine administration interval to improve DBP, but it has the potential for additive benefit. Furthermore, understanding the development of a DBP‐guided chest compression method may advance our understanding of the use and interactions of techniques of physiologic feedback during prolonged resuscitation.

The use of DBP to guide chest compression delivery can be confounded by the response to endogenous or exogenous epinephrine. Previously, we found that when standard CPR was used, DBP measurements during BLS improved during the first 2 minutes of chest compression delivery, plateaued at 3 to 4 minutes, and then decreased without improvement until the initiation of ALS.^21^ We believe that this brief improvement in DBP during the first few minutes of BLS corresponds with distribution of endogenous epinephrine by the initiation of chest compressions and that the effect subsides until the administration of exogenous epinephrine during ALS. These epinephrine‐induced changes in DBP at the beginning of BLS and after epinephrine administration likely need to be anticipated to prevent confusion with improved chest compression delivery. The average DBP during ALS in the DBP‐guided group was 18.4 mm Hg, which is below the AHA's recommended target of 25 to 30 mm Hg in humans^37^ and lower than the baseline DBP in these swine. We believe that this suboptimal DBP likely reflects the prolonged duration of cardiac arrest, as well as the crossover design with artificially short 2‐minute epochs of DBP‐guided chest compression delivery.

The use of a slower compression rate and likely less compression force during DBP‐guided chest compression, as judged by lower mean MAP, mean CVP, and ICP (Table 2), supports the possibility that rapid, forceful compressions generated during ETCO_2_‐guided chest compression limit cardiac filling and therefore DBP.^38^ Similarly, studies of hemodynamic‐directed CPR found that the chest compression depth needed to improve systolic blood pressure was less than that used for standard CPR.^28^, ^29^, ^30^, ^31^, ^32^ By varying the compression rate and force in this neonatal model of asphyxial cardiac arrest, we improved DBP by ≈3 mm Hg using DBP guidance and improved ETCO_2_ by ≈8 mm Hg using ETCO_2_ guidance, changes that were statistically, and maybe clinically, significant. The finding that, in this model, increasing the rate and force of compressions improves ETCO_2_ but possibly at the expense of DBP, and that reduction in rate and force improves DBP but possibly at the expense of ETCO_2_, needs further study and clinical confirmation. With both methods used in this study, the compression rate was greater than that recommended by the AHA for standard CPR.^9^ We did not attempt to compare outcomes, but our previous study found that the faster rate and greater force required to optimize ETCO_2_‐guided chest compression delivery improved short‐term survival over standard CPR in this model.^21^ Outcomes from DBP‐guided chest compression delivery have yet to be examined.

In clinical practice, several physiologic targets may be available for feedback during CPR. A child who has cardiac arrest in the intensive care unit after cardiac surgery often has monitoring that could be used to assess DBP, MPP, ETCO_2_, and cerebral oximetry. Each of these variables could be used as physiologic feedback to assess the quality of resuscitation during prolonged CPR. The DBP and MPP provide information about myocardial perfusion and the likelihood of ROSC, the ETCO_2_ provides information about systemic perfusion and likelihood of response to epinephrine, and the cerebral saturation levels provide information about cerebral perfusion and potential neurologic outcome. Understanding how these 3 vital perfusion pressures are affected by changes to chest compression rate, chest compression force, or epinephrine administration could help optimize outcome from prolonged CPR. We have shown here that optimization of 1 physiologic parameter may result in unexpected, adverse changes to another. Understanding these interactions could be critically important to improving outcomes from prolonged resuscitations, such as during transition onto extracorporeal life support.

The limitations of this study include the preclinical model, though the piglet's chest has a shape, size, and compliance similar to that of a neonate. This anatomy favors the cardiac pump mechanism of blood flow production during CPR and would predict a good response to increased compression rate. This cardiac pump mechanism may be clinically relevant in neonates and infants or during open chest CPR, but the findings related to the use of increased compression rate to optimize ETCO_2_ may not apply to older patients whose chests are less compliant. Additional limitations include the potential for underpowered comparison of some physiologic variables because of the small sample size and the lack of adjustment for multiple comparisons in this pilot study. The use of DBP‐guided chest compression delivery in this study requires vascular access and may only be relevant for patients in the intensive care unit or operating room who have preexisting arterial catheters. The lack of an accelerometer that can be used in a model of this size limited the objective measurement of compression depth or force during the different methods and necessitated that we infer force from pressure measurements. This study did not identify any specific numeric targets for physiologic feedback or outcome data after resuscitation. Rather, it was meant to provide preliminary data to characterize and compare the physiologic changes associated with each of these 2 methods of physiologic feedback. Despite these limitations, our study provokes interest in comparing the use of ETCO_2_ and DBP targets on resuscitation outcomes and in comparing their use with physiologic feedback based on cerebral oximetry.

## Conclusion

In conclusion, use of real‐time physiologic feedback to optimize ETCO_2_ or DBP during resuscitation results in differences in chest compression delivery and clinically relevant parameters, including MPP, ICP, and CPP. Understanding how various methods of physiologic feedback affect hemodynamic parameters is the first step toward developing patient‐centered resuscitation protocols. Future investigation is needed to determine long‐term outcomes, such as survival with a good neurologic outcome, using these physiologic feedback methods.

## Sources of Funding

The authors were supported, in part, by the Eunice Kennedy Shriver National Institute of Child Health and Human Development award R21HD072845, by the National Research Service Award for Clinician Scientists in Pediatric Critical Cardiopulmonary Disease T32HL125239, and by the National Institute of Neurological Disorders and Stroke awards K08NS080984, R21NS095036, and R01NS060703.

## Disclosures

Dr O'Brien's institution received funding from the National Institutes of Health (NIH); she received support for article research from the NIH. Dr Lee received support for article research from the NIH, as well as funding from Medtronic. Dr Koehler received support for article research from the NIH. Dr Hunt received support for article research from the NIH; her institution also received grant funding from the Laerdal Foundation for Acute Care Medicine and from the Hartwell Foundation. Dr Hunt has served as a consultant for Zoll Medical Corporation, receiving honoraria and travel expenses for speaking engagements. Dr Hunt and colleagues have been awarded patents for developing several educational simulation technologies for which Zoll Medical Corporation has a nonexclusive license with the potential for royalties. Dr Shaffner's institution received funding from the National Institute of Child Health and Human Development and the National Institute of Neurological Disorders and Stroke; he received funding from Wolters Kluwer and support for article research from the NIH. The remaining authors have no disclosures to report.

## Supporting information


**Figure S1.** Hemodynamic variables during cardiopulmonary resuscitation. Hemodynamic variables were measured during basic life support (min 0–10) and advanced life support (min 10.5–20) as CPR was delivered with ETCO_2_‐guided chest compression (blue circles) and DBP‐guided chest compression (red squares). Data are presented as mean±SEM at 30‐s intervals. Open circles along the *x*‐axis represent epinephrine administration at 10, 14, and 18 minutes of CPR. A, End‐tidal CO_2_ (ETCO_2_). B, Diastolic blood pressure (DBP). C, Compression rate (CR) in beats per minute (bpm). D, Mean arterial pressure (MAP). E, Mean central venous pressure (mCVP). F, Diastolic central venous pressure (dCVP). G, Systemic perfusion pressure (SPP). H, Myocardial perfusion pressure (MPP). I, Mean intracranial pressure (ICP). J, Cerebral perfusion pressure (CPP).

## References

[1] Gupta P , Tang X , Gall CM , Lauer C , Rice TB , Wetzel RC . Epidemiology and outcomes of in‐hospital cardiac arrest in critically ill children across hospitals of varied center volume: a multi‐center analysis. Resuscitation. 2014;85:1473–1479. 25110249 10.1016/j.resuscitation.2014.07.016

[2] Tibballs J , Kinney S . A prospective study of outcome of in‐patient paediatric cardiopulmonary arrest. Resuscitation. 2006;71:310–318. 17069956 10.1016/j.resuscitation.2006.05.009

[3] Girotra S , Spertus JA , Li Y , Berg RA , Nadkarni VM , Chan PS ; American Heart Association Get with the Guidelines Resuscitation Investigators . Survival trends in pediatric in‐hospital cardiac arrests: an analysis from Get With the Guidelines‐Resuscitation. Circ Cardiovasc Qual Outcomes. 2013;6:42–49. 23250980 10.1161/CIRCOUTCOMES.112.967968 PMC3555689

[4] Moler FW , Meert K , Donaldson AE , Nadkarni V , Brilli RJ , Dalton HJ , Clark RS , Shaffner DH , Schleien CL , Statler K , Tieves KS , Hackbarth R , Pretzlaff R , van der Jagt EW , Levy F , Hernan L , Silverstein FS , Dean JM ; Pediatric Emergency Care Applied Research Network . In‐hospital versus out‐of‐hospital pediatric cardiac arrest: a multicenter cohort study. Crit Care Med. 2009;37:2259–2267. 19455024 10.1097/CCM.0b013e3181a00a6a PMC2711020

[5] Meert KL , Donaldson A , Nadkarni V , Tieves KS , Schleien CL , Brilli RJ , Clark RS , Shaffner DH , Levy F , Statler K , Dalton HJ , van der Jagt EW , Hackbarth R , Pretzlaff R , Hernan L , Dean JM , Moler FW ; Pediatric Emergency Care Applied Research Network . Multicenter cohort study of in‐hospital pediatric cardiac arrest. Pediatr Crit Care Med. 2009;10:544–553. 19451846 10.1097/PCC.0b013e3181a7045c PMC2741542

[6] Del Castillo J , Lopez‐Herce J , Canadas S , Matamoros M , Rodriguez‐Nunez A , Rodriguez‐Calvo A , Carrillo A ; Iberoamerican Pediatric Cardiac Arrest Study Network RIBEPCI . Cardiac arrest and resuscitation in the pediatric intensive care unit: a prospective multicenter multinational study. Resuscitation. 2014;85:1380–1386. 25008138 10.1016/j.resuscitation.2014.06.024

[7] Ortmann L , Prodhan P , Gossett J , Schexnayder S , Berg R , Nadkarni V , Bhutta A ; American Heart Association's Get With the Guidelines Resuscitation Investigators . Outcomes after in‐hospital cardiac arrest in children with cardiac disease: a report from Get With the Guidelines Resuscitation. Circulation. 2011;124:2329–2337. 22025603 10.1161/CIRCULATIONAHA.110.013466

[8] Reis AG , Nadkarni V , Perondi MB , Grisi S , Berg RA . A prospective investigation into the epidemiology of in‐hospital pediatric cardiopulmonary resuscitation using the international Utstein reporting style. Pediatrics. 2002;109:200–209. 11826196 10.1542/peds.109.2.200

[9] de Caen AR , Berg MD , Chameides L , Gooden CK , Hickey RW , Scott HF , Sutton RM , Tijssen JA , Topjian A , van der Jagt EW , Schexnayder SM , Samson RA . Part 12: pediatric advanced life support: 2015 American Heart Association guidelines update for cardiopulmonary resuscitation and emergency cardiovascular care. Circulation. 2015;132:S526–S542. 26473000 10.1161/CIR.0000000000000266 PMC6191296

[10] Gudipati CV , Weil MH , Bisera J , Deshmukh HG , Rackow EC . Expired carbon dioxide: a noninvasive monitor of cardiopulmonary resuscitation. Circulation. 1988;77:234–239. 3121209 10.1161/01.cir.77.1.234

[11] Weil MH , Bisera J , Trevino RP , Rackow EC . Cardiac output and end‐tidal carbon dioxide. Crit Care Med. 1985;13:907–909. 3931979 10.1097/00003246-198511000-00011

[12] Lewis LM , Stothert J , Standeven J , Chandel B , Kurtz M , Fortney J . Correlation of end‐tidal CO2 to cerebral perfusion during CPR. Ann Emerg Med. 1992;21:1131–1134. 1514728 10.1016/s0196-0644(05)80658-4

[13] Falk JL , Rackow EC , Weil MH . End‐tidal carbon dioxide concentration during cardiopulmonary resuscitation. N Engl J Med. 1988;318:607–611. 3125432 10.1056/NEJM198803103181005

[14] Garnett AR , Ornato JP , Gonzalez ER , Johnson EB . End‐tidal carbon dioxide monitoring during cardiopulmonary resuscitation. JAMA. 1987;257:512–515. 3098993

[15] Ornato JP , Garnett AR , Glauser FL . Relationship between cardiac output and the end‐tidal carbon dioxide tension. Ann Emerg Med. 1990;19:1104–1106. 2121075 10.1016/s0196-0644(05)81512-4

[16] Sanders AB , Kern KB , Otto CW , Milander MM , Ewy GA . End‐tidal carbon dioxide monitoring during cardiopulmonary resuscitation. A prognostic indicator for survival. JAMA. 1989;262:1347–1351. 2761035

[17] Callaham M , Barton C . Prediction of outcome of cardiopulmonary resuscitation from end‐tidal carbon dioxide concentration. Crit Care Med. 1990;18:358–362. 2108000 10.1097/00003246-199004000-00002

[18] Grmec S , Klemen P . Does the end‐tidal carbon dioxide (EtCO2) concentration have prognostic value during out‐of‐hospital cardiac arrest? Eur J Emerg Med. 2001;8:263–269. 11785591 10.1097/00063110-200112000-00003

[19] Kalenda Z . The capnogram as a guide to the efficacy of cardiac massage. Resuscitation. 1978;6:259–263. 755268 10.1016/0300-9572(78)90006-0

[20] Sutton RM , French B , Meaney PA , Topjian AA , Parshuram CS , Edelson DP , Schexnayder S , Abella BS , Merchant RM , Bembea M , Berg RA , Nadkarni VM ; American Heart Association's Get With The Guidelines Resuscitation Investigators . Physiologic monitoring of CPR quality during adult cardiac arrest: a propensity‐matched cohort study. Resuscitation. 2016;106:76–82. 27350369 10.1016/j.resuscitation.2016.06.018 PMC4996723

[21] Hamrick JT , Hamrick JL , Bhalala U , Armstrong JS , Lee JH , Kulikowicz E , Lee JK , Kudchadkar SR , Koehler RC , Hunt EA , Shaffner DH . End‐tidal CO2‐guided chest compression delivery improves survival in a neonatal asphyxial cardiac arrest model. Pediatr Crit Care Med. 2017;18:e574–e578. 10.1097/PCC.0000000000001299 PMC5669831 28817508

[22] Niemann JT , Criley JM , Rosborough JP , Niskanen RA , Alferness C . Predictive indices of successful cardiac resuscitation after prolonged arrest and experimental cardiopulmonary resuscitation. Ann Emerg Med. 1985;14:521–528. 3994075 10.1016/s0196-0644(85)80774-5

[23] Kern KB , Ewy GA , Voorhees WD , Babbs CF , Tacker WA . Myocardial perfusion pressure: a predictor of 24‐hour survival during prolonged cardiac arrest in dogs. Resuscitation. 1988;16:241–250. 2849790 10.1016/0300-9572(88)90111-6

[24] Reynolds JC , Salcido DD , Menegazzi JJ . Coronary perfusion pressure and return of spontaneous circulation after prolonged cardiac arrest. Prehosp Emerg Care. 2010;14:78–84. 19947871 10.3109/10903120903349796 PMC2922866

[25] Sanders AB , Kern KB , Atlas M , Bragg S , Ewy GA . Importance of the duration of inadequate coronary perfusion pressure on resuscitation from cardiac arrest. J Am Coll Cardiol. 1985;6:113–118. 4008767 10.1016/s0735-1097(85)80261-8

[26] Paradis NA , Martin GB , Rivers EP , Goetting MG , Appleton TJ , Feingold M , Nowak RM . Coronary perfusion pressure and the return of spontaneous circulation in human cardiopulmonary resuscitation. JAMA. 1990;263:1106–1113. 2386557

[27] Morgan RW , French B , Kilbaugh TJ , Naim MY , Wolfe H , Bratinov G , Shoap W , Hsieh TC , Nadkarni VM , Berg RA , Sutton RM . A quantitative comparison of physiologic indicators of cardiopulmonary resuscitation quality: diastolic blood pressure versus end‐tidal carbon dioxide. Resuscitation. 2016;104:6–11. 27107688 10.1016/j.resuscitation.2016.04.004 PMC4902744

[28] Sutton RM , Friess SH , Naim MY , Lampe JW , Bratinov G , Weiland TR III , Garuccio M , Nadkarni VM , Becker LB , Berg RA . Patient‐centric blood pressure‐targeted cardiopulmonary resuscitation improves survival from cardiac arrest. Am J Respir Crit Care Med. 2014;190:1255–1262. 25321490 10.1164/rccm.201407-1343OC PMC4315818

[29] Friess SH , Sutton RM , Bhalala U , Maltese MR , Naim MY , Bratinov G , Weiland TR III , Garuccio M , Nadkarni VM , Becker LB , Berg RA . Hemodynamic directed cardiopulmonary resuscitation improves short‐term survival from ventricular fibrillation cardiac arrest. Crit Care Med. 2013;41:2698–2704. 23887237 10.1097/CCM.0b013e318298ad6b PMC3812371

[30] Sutton RM , Friess SH , Bhalala U , Maltese MR , Naim MY , Bratinov G , Niles D , Nadkarni VM , Becker LB , Berg RA . Hemodynamic directed CPR improves short‐term survival from asphyxia‐associated cardiac arrest. Resuscitation. 2013;84:696–701. 23142199 10.1016/j.resuscitation.2012.10.023 PMC3612383

[31] Morgan RW , Kilbaugh TJ , Shoap W , Bratinov G , Lin Y , Hsieh TC , Nadkarni VM , Berg RA , Sutton RM ; Pediatric Cardiac Arrest Survival Outcomes PiCASO Laboratory Investigators . A hemodynamic‐directed approach to pediatric cardiopulmonary resuscitation (HD‐CPR) improves survival. Resuscitation. 2017;111:41–47. 27923692 10.1016/j.resuscitation.2016.11.018 PMC5218511

[32] Naim MY , Sutton RM , Friess SH , Bratinov G , Bhalala U , Kilbaugh TJ , Lampe JW , Nadkarni VM , Becker LB , Berg RA . Blood pressure‐ and coronary perfusion pressure‐targeted cardiopulmonary resuscitation improves 24‐hour survival from ventricular fibrillation cardiac arrest. Crit Care Med. 2016;44:e1111–e1117. 27414479 10.1097/CCM.0000000000001859 PMC5069077

[33] Neurauter A , Nysaether J , Kramer‐Johansen J , Eilevstjonn J , Paal P , Myklebust H , Wenzel V , Lindner KH , Schmolz W , Pytte M , Steen PA , Strohmenger HU . Comparison of mechanical characteristics of the human and porcine chest during cardiopulmonary resuscitation. Resuscitation. 2009;80:463–469. 19195761 10.1016/j.resuscitation.2008.12.014

[34] Hamrick JL , Hamrick JT , Lee JK , Lee BH , Koehler RC , Shaffner DH . Efficacy of chest compressions directed by end‐tidal CO2 feedback in a pediatric resuscitation model of basic life support. J Am Heart Assoc. 2014;3:e000450 DOI: 10.1161/JAHA.113.000450. 24732917 PMC4187472

[35] Berg RA , Henry C , Otto CW , Sanders AB , Kern KB , Hilwig RW , Ewy GA . Initial end‐tidal CO2 is markedly elevated during cardiopulmonary resuscitation after asphyxial cardiac arrest. Pediatr Emerg Care. 1996;12:245–248. 8858644 10.1097/00006565-199608000-00002

[36] Grmec S , Lah K , Tusek‐Bunc K . Difference in end‐tidal CO2 between asphyxia cardiac arrest and ventricular fibrillation/pulseless ventricular tachycardia cardiac arrest in the prehospital setting. Crit Care. 2003;7:R139–144. 14624688 10.1186/cc2369 PMC374361

[37] Meaney PA , Bobrow BJ , Mancini ME , Christenson J , de Caen AR , Bhanji F , Abella BS , Kleinman ME , Edelson DP , Berg RA , Aufderheide TP , Menon V , Leary M ; CPR Quality Summit Investigators, American Heart Association Emergency Cardiovascular Care Committee, Council on Cardiopulmonary Critical Care and Perioperative Resuscitation . Cardiopulmonary resuscitation quality: [corrected] improving cardiac resuscitation outcomes both inside and outside the hospital: a consensus statement from the American Heart Association. Circulation. 2013;128:417–435. 23801105 10.1161/CIR.0b013e31829d8654

[38] Maier GW , Tyson GS Jr , Olsen CO , Kernstein KH , Davis JW , Conn EH , Sabiston DC Jr , Rankin JS . The physiology of external cardiac massage: high‐impulse cardiopulmonary resuscitation. Circulation. 1984;70:86–101. 6723014 10.1161/01.cir.70.1.86

