# Supplementary material for: Pilot Study to Compare the Use of End‐Tidal Carbon Dioxide–Guided and Diastolic Blood Pressure–Guided Chest Compression Delivery in a Swine Model of Neonatal Asphyxial Cardiac Arrest
Source: J Am Heart Assoc. 2018 Sep 27;7(19):e009728. doi: 10.1161/JAHA.118.009728 (PMC6404892; doi:10.1161/JAHA.118.009728)
Supplement: Supplementary file 1 — Figure S1. Hemodynamic variables during cardiopulmonary resuscitation. Hemodynamic variables were measured during basic life support (min 0–10) and advanced life support (min 10.5–20) as CPR was delivered with ETCO2‐guided chest compression (blue circles) and DBP‐guided chest compression (red squares). Data are presented as mean±SEM at 30‐s intervals. Open circles along the x‐axis represent epinephrine administration at 10, 14, and 18 minutes of CPR. A, End‐tidal CO2 (ETCO2). B, Diastolic blood pressure (DBP). C, Compression rate (CR) in beats per minute (bpm). D, Mean arterial pressure (MAP). E, Mean central venous pressure (mCVP). F, Diastolic central venous pressure (dCVP). G, Systemic perfusion pressure (SPP). H, Myocardial perfusion pressure (MPP). I, Mean intracranial pressure (ICP). J, Cerebral perfusion pressure (CPP). [file JAH3-7-e009728-s001.pdf]

Supplemental Material

Figure S1. Hemodynamic variables during cardiopulmonary resuscitation.

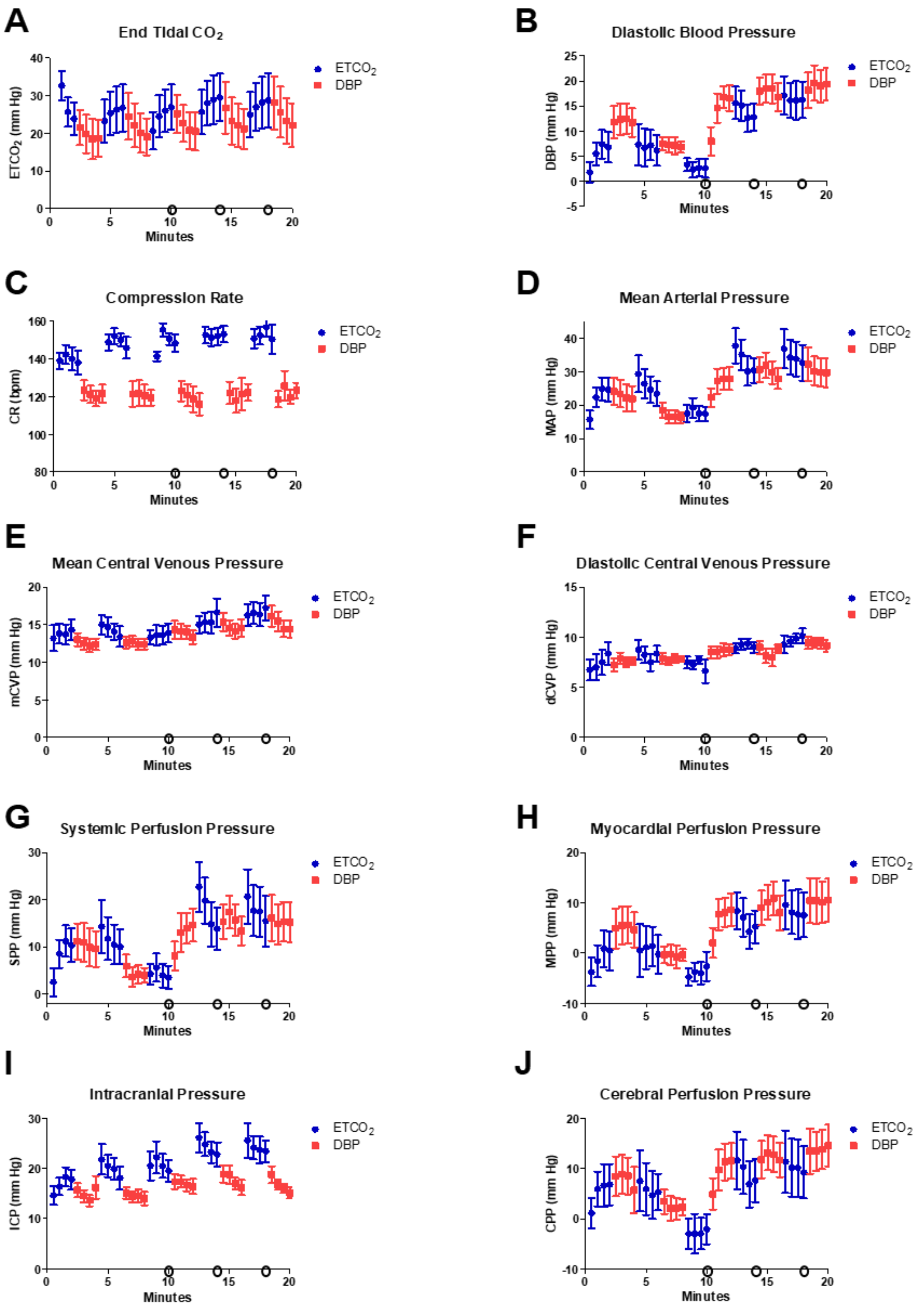

Hemodynamic variables were measured during basic life support (min 0-10) and advanced life support (min 10.5-20) as CPR was delivered with $ETCO_2$-guided chest compression (blue

circles) and DBP-guided chest compression (red squares). Data are presented as mean ± standard error of the mean at 30-second intervals. Open circles along the x-axis represent epinephrine administration at 10, 14, and 18 min of CPR. **A**, End-tidal CO_2 ($ETCO_2$). **B**, Diastolic blood pressure (DBP). **C**, Compression rate (CR) in beats per minute (bpm). **D**, Mean arterial pressure (MAP). **E**, Mean central venous pressure (mCVP). **F**, Diastolic central venous pressure (dCVP). **G**, Systemic perfusion pressure (SPP). **H**, Myocardial perfusion pressure (MPP). **I**, Mean intracranial pressure (ICP). **J**, Cerebral perfusion pressure (CPP).
